# Short interpregnancy interval and low birth weight births in India: Evidence from National Family Health Survey 2015-16

**DOI:** 10.1016/j.ssmph.2020.100700

**Published:** 2020-11-24

**Authors:** Ajit Kumar Kannaujiya, Kaushalendra Kumar, Ashish Kumar Upadhyay, Lotus McDougal, Anita Raj, Abhishek Singh

**Affiliations:** a International Institute for Population Sciences, Mumbai, India; b Department of Public Health & Mortality Studies, International Institute for Population Sciences, Mumbai, India; c Center on Gender Equity and Health, University of California San Diego, United States

**Keywords:** Interpregnancy interval, Low birth weight, NFHS 2015-16, Reproductive calendar, Multivariable binary logistic regression, India

## Abstract

Evidence on the effect of interpregnancy interval (IPI) on low birth weight (LBW) births is limited in developing countries including India. Our study aims to examine association between IPI and LBW births in India. We used data from the fourth round of the National Family Health Survey (NFHS-4) conducted in 2015–16 with a representative sample of 52,825 most recent births for examining the association between IPI and LBW. IPI is defined as the gap between the first month in which the index pregnancy was reported in the reproductive calendar (referred to as the month of conception) and the month of pregnancy outcome (including live births and terminations) of preceding pregnancy. Reproductive calendar data were used to estimate IPI. Association between IPI and LBW were examined using multivariable binary logistic regressions. Seventeen percent of the births in our sample were LBW, and more than half (57.6%) of these were accompanied with IPI less than 18 months. Prevalence of LBW births was highest among mother's who had IPI less than six months (19.4%). Regression results, adjusted for control variables, indicate that the risk of LBW was significantly higher among births whose mothers had IPI less than six months (odds ratio: 1.19, 95% CI:1.05-1.36) compared with those whose mothers had IPI between 18 and 23 months. This study provides additional evidence on the association between short IPI (<6 months) and LBW births in India. Promoting spacing methods of family planning is an option that India may consider for increasing the IPI and thereby reducing LBW births. Ensuring recommended iron and folic acid tablets/equivalent syrup and TT injections for every pregnant woman may offset the adverse consequences of shorter IPI.

## Highlights

• 12% of the LBW births were accompanied with IPI <6 months, 20% between 6 and 11 months, and 25% between 12 and 17 months.
• The risk of LBW births is higher among mothers having an IPI of <6 months compared with mothers having an IPI of 18–23 months.
• Promoting spacing methods of family planning may consider for increasing the IPI and thereby reducing LBW births in India.
• Promoting recommended TT injections and iron-folic acid supplements may offset the adverse consequences of short IPI.

## 1 Introduction

Low birth weight (LBW), generally defined as a birth weight less than 2500 g, is associated with a range of both short- and long-term consequences affecting human capital (Katz et al., 2013; WHO & UNICEF, 2004). Studies show that more than 80% of neonatal deaths are in LBW newborns (Katz et al., 2013; Lawn et al., 2014; Lee et al., 2013). LBW is also linked with higher risk of childhood morbidity, stunting in childhood, and long-term developmental and physical ill-health including chronic illnesses such as cardiovascular diseases (Blencowe et al., 2013; Christian et al., 2013; Gluckman, Hanson, & Beedle, 2007).

Recent estimates from the World Health Organization suggest that about 15–20% of all births worldwide are LBW, representing more than 20 million births in a year. Of these, almost 28% belong to South Asian countries (WHO, 2014). India also has a considerable burden of LBW births, with recent estimates suggesting that 18% of births in 2015–16 were LBW (IIPS & ICF, 2017). Moreover, various states of India are marked by considerable inequalities; prevalence of LBW ranges between 6% and 27% across the Indian states (IIPS & ICF, 2017).

**Fig. 1:**
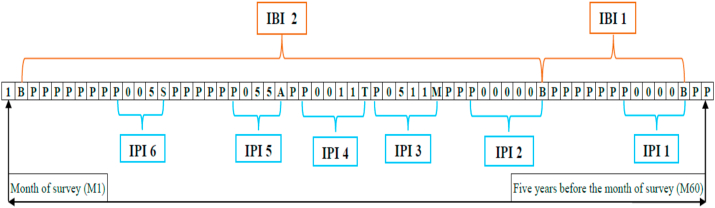
Pictorial representation of IPI. P – pregnant, B – Birth, S – Still birth, A – Abortion, T – Termination, M – Miscarriage, 0 – not using any contraception, 1– using pill, 5 – using condom. Note: One box represents one month.

Past studies have used a number of indicators related to spacing between pregnancies or births – interbirth intervals (IBI), interpregnancy intervals (IPI), inter-outcome intervals (IOI) - to explain adverse pregnancy outcomes. IPI is defined as the gap between the first month the index pregnancy was reported in the reproductive calendar (referred to as the month of conception) and the month of pregnancy outcome (including live births and terminations) for the second-most-recent pregnancy (Klerman, Cliver, & Goldenberg, 1998; WHO, 2007; Cecatti, Correa-Silva, Milanez, Morais, & Souza, 2008; Eleje, Ezebialu, & Eke, 2011; Davis et al., 2014). On the other hand, IBI is defined as the interval between two consecutive births. IOI is simply the sum of IPI and the duration of gestation of the index pregnancy (DaVanzo et al., 2004). As opposed to IBI, IPI is likely to provide more robust association with adverse pregnancy outcomes as IBI ignores those pregnancies that resulted in adverse pregnancy outcomes such as miscarriage, abortion, and stillbirths between these two consecutive live births (Fig. 1). Since IOI is the sum of IPI and duration of gestation of the index pregnancy, the estimated effect of IOI would be the same as the effect of IPI (DaVanzo et al., 2004). Recent data from India suggests an average IPI of 16.7 months in 2015–16, with no change in the past 10 years (IIPS & ICF, 2007, 2017). Average IPI in India is much shorter than in countries with similar or lower levels of socio-economic development (Cecatti et al., 2008; Mahande & Obure, 2016; Mignini et al., 2016).

Although the prevalence of LBW births is considerable in India, the causes of LBW births are not fully understood. There are a range of sociodemographic factors and health care utilization measures that are associated with LBW births. Some of the sociodemographic and health care utilization factors associated with LBW births in India are lower maternal age, poor maternal nutrition, lower maternal height, lower socioeconomic status, exposure to passive smoking, late registration for antenatal care, lack of recommended antenatal visits, etc. (Chakraborty, Roy, & Das, 1975; Dharmalingam, Navaneetham, & Krishnakumar, 2010; Kader & Perera, 2014; Mumbare et al., 2012). Higher birth order and female sex of the child are also found to be associated with LBW births in a few studies (Kader & Perera, 2014; Khan, Mozumdar, & Kaur, 2020; Taywade & Pisudde, 2017). IPI is a factor that has been identified as an important precursor of LBW births in other developed and a few developing countries such as United States of America (USA), Canada, Poland, Latin American countries, and Tanzania (Adams, Delaney, Stupp, McCarthy, & Rawlings, 1997; Miller, 1994; Zhu & Le, 2003), possibly through maternal nutritional depletion, maternal folate depletion, vertical transmission of infections and cervical insufficiency (Conde-Agudelo, Rosas-Bermudez, Castano, & Norton, 2012). These studies are based primarily on livebirth certificates, vital statistics or birth registry. Unfortunately, this relationship is not well understood in India. Only two studies from India have explored this relationship (Kader & Perera, 2014; Mavalankar, Gray, & Trivedi, 1992). Kader and Perera (2014), using National Family Health Survey 2005-06, found no association between short IPI and LBW births in India (Kader & Perera, 2014). A key limitation of Kader and Perera (2014) is their broad categorization of IPI (<18 months, 18–59 months, and >59 months). Additionally, these data are from more than a decade ago and may be less reflective of current realities. Mavalankar et al. (1992), using a hospital-based case-control study linked with a population survey in Ahmedabad India, reported an association between short IPI and increased risk of preterm LBW birth (Mavalankar et al., 1992), but again, these data are dated.

Having identified the afore-mentioned gaps in the literature, our study examines the association between IPI and LBW births in India, a large and diverse country, using reproductive calendar data collected in the most recent round of National Family Health Survey 2015-16, henceforth referred to as NFHS-4.

## 2 Materials and methods

### 2.1 Data

We used data from NFHS-4 conducted in 29 states and 7 Union Territories (UTs) of India. NFHS-4 provides information on maternal and child health, family planning, other reproductive health indicators as well as sexual behavior; HIV/AIDS knowledge, attitudes, and behavior; and domestic violence (IIPS & ICF, 2017). A total of 699,686 eligible women age 15–49 were interviewed with a response rate of 97% (IIPS & ICF, 2017).

#### 2.1.1 Analytical sample

We used reproductive calendar collected in NFHS-4 to calculate the IPI. The reproductive calendar includes a monthly history of key events such as births, pregnancies, pregnancy terminations, contraceptive use, and reasons for discontinuation of contraception use for a period going back 60 months before the survey. We restricted our sample to those women who had reported at least two pregnancies in the reproductive calendar, with the most recent of those births having occurred in five years preceding the survey. This time frame was necessary as information on birth weight is available for only those births that occurred in five years preceeding the survey. Of the total 699,686 women interviewed in NFHS-4, 83,166 women reported at least two pregnancies in the reproductive calendar. Of these, we excluded 10,096 women who reported non-live births for their most recent pregnancy. We excluded an additional 21 women who reported pregnancies with durations greater than 10 months. Moreover, 19,789 births were excluded because of the unavailability of birthweight. Finally, 435 twin births were excluded from the sample. Following these inclusion and exclusion criteria, our analytical sample is limited to 52,825 most recent singleton births. Notably, our unit of analysis is the most recent birth within the past five years, because information on antenatal visits, TT injections, and IFA tablets/equivalent syrup were collected only in reference to the most recent birth. Therefore, each woman in our sample contributes only one IPI. Hence number of women and children are same in our sample.

#### 2.1.2 Dependent variable

The dependent variable used in this analysis is LBW, defined as weight below 2500 g at birth.

Information on birth weight was collected by asking the following questions to recent mothers:

1) “Was (NAME) weighed at birth?” if “Yes” then
2) (a) “How much did (NAME) weigh?” and have (b) “Record weight in kilograms from health card, if available.” If the health card was not available, birth weight was recorded as per mother's recall.

Children who weighed less than 2500 g at birth were coded as LBW (‘1’) and remaining were coded as normal birth weight (‘0’).

#### 2.1.3 Independent variable

The key independent variable of interest is IPI which is defined as the gap between the first month the index pregnancy was reported in the reproductive calendar (referred to as the month of conception) and the month of pregnancy outcome (including live births and terminations) of the preceding pregnancy (Fig. 1). IPI was categorized into nine groups - <6, 6–11, 12–17, 18–23, 24–29, 30–35, 36–41, 42–47, and ≥48 months. Available literature suggests an IPI of 18–23 months to minimize the risk of adverse maternal and child health outcomes (Conde-Agudelo, Rosas-Bermúdez, & Kafury-Goeta, 2006; Zhu & Le, 2003). In the light of existing literature, we coded IPI of 18–23 months as the reference category in the regression models.

#### 2.1.4 Control variables

Based on previous studies on LBW births in India and abroad, a number of maternal-level, child-level, maternal and child-care program-level and household-level variables were included. Maternal-level variables are: mother's age at marriage (<18 years, 18–24 years, 25–29 years, ≥30 years), mother's age at conception (<20 years, 20–24 years, 25–29 years, ≥30 years), mother's height (<145 cm, ≥145 cm), mother's schooling (no schooling, primary, secondary, higher), mother smokes tobacco (no, yes), outcome of previous pregnancy (birth, abortion, miscarriage/termination and stillbirth of the pregnancy prior to the index pregnancy), postpartum contraceptive use after previous pregnancy outcome (no, yes). Child-level variables include: birth order (1, 2, 3, ≥4), index birth wanted (no, yes), index birth preterm (no, yes). Maternal and child care program variables include: ≥4 antenatal care (ANC) visits (no, yes), ≥2 Tetanus Toxoid (TT) injections (no, yes), consumed ≥100 Iron Folic Acid (IFA) tablets/equivalent syrup (no, yes). Household-level variables include: wealth quintiles (poorest, poorer, middle, richer, richest), religion (Hindu, Muslim, Christian, others), caste (scheduled caste, scheduled tribe, other backward class, others), geographical region (north, central, east, northeast, west, south), urban-rural residence (urban, rural).

Postpartum contraceptive use refers to the use of contraceptive methods between outcome of previous pregnancy and the next pregnancy. Reproductive calendar records monthly information of the use of contraceptive method used by women. Women who reported using any method were coded as ‘yes’ and rest were coded as ‘no’.

Index birth wanted was created using the following two questions canvassed in NFHS-4:

When you got pregnant with (Name), did you want to get pregnant at that time? (Yes, No).

If the women answered no, then NFHS-4 further asked.

Did you want to have a baby later on, or did you not want any (more) children? (Later, No more) Births for whom the mothers answered ‘no more’ were coded as not wanted. Rest of the births were coded as wanted.

NFHS-4 also collected information on antenatal care received in reference to the most recent birth. NFHS-4 asked women.

During this pregnancy, were you given or did you buy any iron folic acid tablets or syrup? (Yes, No, Don't know).

If the women reported yes, then NFHS-4 further asked.

During the whole pregnancy, for how many days did you take the tablets or syrup?

Mothers who reported taking tablets/syrup for 100 or more days were coded as consumed ≥100 IFA tablets/syrup and rest were coded as not consumed ≥100 IFA tablets/syrup.

NFHS-4 also collected information on TT injections received by women during their last pregnancy. Women who received 2 or more TT injections were coded as having received 2 or more TT injections and rest were coded as not having received 2 or more TT injections.

## 2.2 Methods

As the dependent variable is binary, we used a multivariable binary logistic regression model to examine the association between IPI and LBW adjusting for other variables listed in the previous section. We estimated a series of regression models to examine the association between IPI and LBW in India. In model-1, we regressed LBW on IPI. In model 2, we added maternal-level variables along with IPI. In model 3, we added child-level variables along with independent variables included in model 2. Similarly, we added maternal and child care program related variables in model 4 and household-level variables in model 5. Odds ratios and 95% confidence intervals were reported. We also tested for interactions between education and other strongly associated covariates (e.g. interaction of education with TT and IFA tablets/syrup). However, as the results did not change, we opted to not include interactions in the final models. Since NFHS-4 used a multistage sampling design, appropriate sampling weights were used in estimations. The details of the sampling weights are given in the NFHS-4 report (IIPS & ICF, 2017). Appropriate adjustments were also made for the complex survey design employed in NFHS-4. All the statistical analysis were conducted in Stata 13.1.

## 3 Results

### 3.1 Descriptive results

Seventeen percent of the most recent births in our sample were LBW. More than half of these births (57.6%) were accompanied with IPI less than 18 months (Table 1). Of these, 12% were accompanied with IPI less than six months, 20% between 6 and 11 months, and 25% between 12 and 17 months. Only 18% of the births were accompanied with IPI between 18 and 23 months. One-fourth births were accompanied with IPI of 24 months and above. Six percent of the births were preterm and 11% were unwanted. Mothers of about 53% of births received the recommended four or more ANC visits, 83% received two or more TT injections, and 33% consumed 100 or more IFA tablets/equivalent syrup during their most recent pregnancy. Mothers of fourteen percent of births experienced an adverse pregnancy outcome such as abortion (3.1%), miscarriage/termination (10.0%), and stillbirth (1.5%) for their second-to-last pregnancy.

**Table 1:** Percent distribution of independent and control variables, and prevalence of low birth weight by the independent and control variables, India, NFHS-4 (2015-16).

Characteristics	Sample (N)^1^	Percent Distribution^2^	Prevalence of low birth weight^2^	P value
Total	52,825	100	17.1	
**Interpregnancy interval**				
<6	6211	12.1	19.4	<0.001
06–11	10,088	20.2	17.2	
12–17	13,178	25.3	17.4	
18–23	9456	17.6	16.0	
24–29	6387	11.6	16.7	
30–35	3753	6.8	16.6	
36–41	2219	3.9	15.8	
42–47	1089	1.9	16.3	
≥48	444	0.7	18.5	
**Mother's age at marriage**				
<18 years	16,829	33.9	18.2	<0.001
18–24 years	31,599	59.4	16.5	
25–29 years	3714	5.9	17.3	
≥30 years	683	0.8	19.2	
**Mother's age at conception**				
<20 years	3362	7.7	19.2	<0.001
20–24 years	24,658	49.2	17.1	
25–29 years	17,278	31.4	16.6	
≥30 years	7527	11.6	17.5	
**Mother's height**				
<145 cm	6008	11.6	21.0	<0.001
≥145 cm	46,817	88.4	16.6	
**Mother's schooling**				
No schooling	14,643	26.5	19.1	<0.001
Primary	8109	14.9	18.3	
Secondary	25,652	49.5	16.5	
Higher	4421	9.1	12.9	
**Mother smokes tobacco**				
No	47,760	94.6	17.0	0.044
Yes	5065	5.4	19.5	
**Outcome of previous pregnancy**				
Birth	45,105	85.4	17.0	0.01
Abortion	1527	3.1	16.9	
Miscarriage/termination	5391	10.0	18.2	
Stillbirth	802	1.5	19.0	
**Postpartum contraceptive use after previouspregnancyoutcome**				
No	44,590	85.1	17.3	0.323
Yes	8235	14.9	16.3	
**Birth order**				
1	3591	7.0	18.9	<0.001
2	27,589	54.9	17.0	
3	12,106	22.4	16.3	
≥4	9539	15.7	18.2	
**Index birth wanted**				
No	5525	10.8	17.7	<0.001
Yes	47,300	89.2	17.1	
**Index birth preterm**				
No	49,606	93.9	16.4	<0.001
Yes	3219	6.1	29.1	
**≥4 ANC visits**				
No	26,695	46.9	17.9	<0.001
Yes	26,130	53.1	16.4	
**≥2 TT injections**				
No	9158	16.9	19.7	<0.001
Yes	43,667	83.1	16.6	
**Consumed ≥100 IFA tablets/equivalent syrup**				
No	36,977	67.0	18.2	<0.001
Yes	15,848	33.0	15.0	
**Wealth quintiles**				
Poorest	12,440	22.1	18.1	<0.001
Poorer	12,705	22.9	17.2	
Middle	11,366	21.8	17.6	
Richer	9423	19.4	17.2	
Richest	6891	13.8	14.7	
**Religion**				
Hindu	39,528	79.7	17.4	<0.001
Muslim	7746	15.5	15.9	
Christian	3645	2.0	14.7	
Others	1906	2.8	17.3	
**Caste**				
Scheduled Caste	10,383	21.8	18.2	<0.001
Scheduled Tribe	9793	10.5	18.2	
Other Backward Class	21,415	44.4	16.6	
Others	11,234	23.3	16.7	
**Geographical Region**				
North	10,251	13.5	21.4	<0.001
Central	14,632	23.7	19.2	
East	10,881	22.8	14.0	
Northeast	6148	3.0	13.0	
West	4457	15.0	18.4	
South	6456	22.1	15.2	
**Urban-rural residence**				
Urban	12,678	28.5	17.1	0.195
Rural	40,147	71.5	17.2	

**Note:** 1. N's are unweighted, 2. Weighted percentages.

The prevalence of LBW was highest among births whose mothers had IPI less six months (19.4%) followed by births whose mothers had IPI more than 48 months (18.5%) (Table 1). Prevalence of LBW was lowest among births whose mothers had IPI between 36 and 41 months (15.8%). Prevalence of LBW was higher among births of mothers who conceived before the age of 20 (19.2%), had height below 145 cm (cms.) (21.0%), and had no schooling (19.1%). Prevalence of LBW was also higher among births whose mothers had experienced an adverse pregnancy outcome for their second-to-last pregnancy. The prevalence of LBW was also higher among preterm (29.1%) and unwanted births (17.7%). Prevalence of LBW was lower among mothers those who had the recommended four or more ANC visits, received two or more TT injections, consumed 100 or more IFA tablets/equivalent syrup; and did not report consuming tobacco. Prevalence of LBW was also lower among mothers who lived in urban areas; were from richest wealth quintile, followed Christian religion, and belonged to other backward class.

### 3.2 Multivariable regression results

Results of logistic regression models to examine the association between IPI and LBW are shown in Table 2. IPI less than six months was significantly associated with LBW births in the model 1 (odds ratio: 1.26, 95% CI: 1.12 to 1.41). Although the odds ratio declined marginally in model 2, the statistical significance remained. The odds ratio did not change when we added child-level variables and maternal and child care program related variables in models 3 and 4 respectively. The odds ratio declined to 1.19 when we added household-related variables (see model 5), though statistical significance remained unchanged. In model 5, the risk of LBW was 1.19 (95% CI:1.05 to 1.36) times higher among births whose mothers had an IPI of less than six months compared with births whose mothers had an IPI of 18–23 months.

**Table 2:** Odds ratio of being low birth weight among most recent singleton births, India, NFHS-4 (2015–16).

Characteristics	Odds Ratio (95% CI)	Odds Ratio (95% CI)	Odds Ratio (95% CI)	Odds Ratio (95% CI)	Odds Ratio (95% CI)
	Model-1	Model-2	Model-3	Model-4	Model-5
**Interpregnancy interval**					
<6	1.26*(1.12,1.41)	1.24*(1.09,1.40)	1.24*(1.1,1.41)	1.24*(1.09,1.40)	1.19*(1.05,1.36)
06–11	1.09(0.97,1.21)	1.08(0.97,1.21)	1.09(0.98,1.22)	1.09(0.98,1.22)	1.07(0.95,1.19)
12–17	1.10(0.99,1.21)	1.09(0.99,1.21)	1.10(0.99,1.21)	1.10(0.99,1.21)	1.08(0.98,1.19)
18-23 ®	1.00	1.00	1.00	1.00	1.00
24–29	1.05(0.93,1.18)	1.05(0.93,1.18)	1.03(0.92,1.17)	1.04(0.92,1.17)	1.05(0.93,1.18)
30–35	1.04(0.85,1.28)	1.05(0.86,1.28)	1.04(0.84,1.27)	1.04(0.85,1.28)	1.04(0.86,1.27)
36–41	0.98(0.82,1.16)	1.00(0.84,1.19)	0.99(0.83,1.18)	0.99(0.83,1.18)	1.02(0.86,1.22)
42–47	1.02(0.80,1.30)	1.06(0.82,1.35)	1.07(0.83,1.37)	1.08(0.84,1.38)	1.12(0.87,1.44)
≥48	1.19(0.79,1.79)	1.20(0.80,1.79)	1.14(0.77,1.71)	1.16(0.78,1.73)	1.28(0.85,1.92)
**Mother's** a**ge at marriage**					
<18		1.05(0.97,1.13)	1.07(0.98,1.16)	1.06(0.98,1.15)	1.06(0.98,1.15)
18-24®		1.00	1.00	1.00	1.00
25–29		1.17(0.99,1.37)	1.14(0.97,1.34)	1.14(0.97,1.34)	1.17(0.99,1.38)
≥30		1.30(0.90,1.89)	1.22(0.83,1.80)	1.23(0.84,1.81)	1.33(0.90,1.95)
**Mother's age at conception**					
<20 years		1.08(0.94,1.23)	1.05(0.92,1.21)	1.05(0.92,1.21)	1.10(0.96,1.27)
20–24 years ®		1.00	1.00	1.00	1.00
25–29 years		0.97(0.90,1.05)	0.99(0.91,1.08)	0.99(0.91,1.08)	0.98(0.90,1.07)
≥30 years		0.96(0.86,1.08)	0.99(0.87,1.12)	0.99(0.87,1.13)	0.98(0.86,1.12)
**Mother's height**					
<145 cm		1.28*(1.16,1.41)	1.27*(1.15,1.41)	1.27*(1.15,1.41)	1.34*(1.21,1.48)
≥145 cm ®		1.00	1.00	1.00	1.00
**Mother's Schooling**					
No schooling		1.59*(1.37,1.85)	1.65*(1.41,1.93)	1.57*(1.35,1.84)	1.53*(1.30,1.81)
Primary		1.51*(1.29,1.78)	1.58*(1.34,1.87)	1.53*(1.29,1.81)	1.46*(1.23,1.75)
Secondary		1.34*(1.16,1.55)	1.38*(1.19,1.60)	1.35*(1.17,1.57)	1.32*(1.13,1.54)
Higher ®		1.00	1.00	1.00	1.00
**Mother** s**mokestobacco**					
No ®		1.00	1.00	1.00	1.00
Yes		1.11(0.97,1.27)	1.11(0.97,1.27)	1.11(0.97,1.27)	1.09(0.94,1.25)
**Previous pregnancy outcome**					
Birth ®		1.00	1.00	1.00	1.00
Abortion		1.01(0.84,1.23)	0.97(0.80,1.19)	0.99(0.81,1.20)	1.03(0.85,1.26)
Miscarriage/termination		1.09(0.97,1.21)	1.00(0.87,1.15)	1.01(0.88,1.15)	1.02(0.89,1.17)
Still birth		1.08(0.85,1.37)	0.99(0.77,1.28)	0.99(0.77,1.29)	1.03(0.80,1.33)
**Postpartum contraceptive useafter previous pregnancy outcome**					
No		1.02(0.93,1.13)	1.01(0.92,1.11)	0.99(0.90,1.10)	1.02(0.92,1.12)
Yes ®		1.00	1.00	1.00	1.00
**Birth order**					
1			1.16(0.94,1.42)	1.19(0.97,1.46)	1.18(0.97,1.45)
2			1.05(0.94,1.16)	1.06(0.96,1.19)	1.05(0.95,1.17)
3			0.93(0.84,1.03)	0.94(0.85,1.04)	0.94(0.84,1.04)
≥4®			1.00	1.00	1.00
**Indexbirthwanted**					
No			1.04(0.95,1.15)	1.01(0.92,1.12)	1.04(0.94,1.14)
Yes ®			1.00	1.00	1.00
**Index birth preterm**					
No ®			1.00	1.00	1.00
Yes			2.12*(1.89,2.38)	2.11*(1.88,2.37)	2.16*(1.92,2.42)
**≥4 ANC Visits**					
No				0.99(0.93,1.07)	1.01(0.93,1.08)
Yes ®				1.00	1.00
**≥2 TT injections**					
No				1.22*(1.12,1.32)	1.20*(1.11,1.31)
Yes ®				1.00	1.00
**Consumed≥100 day IFA tablets/equivalentsyrup**					
No				1.19*(1.09,1.30)	1.18*(1.09,1.29)
Yes ®				1.00	1.00
**Wealth Quintiles**					
Poorest					1.29*(1.10,1.51)
Poorer					1.21*(1.04,1.40)
Middle					1.24*(1.07,1.43)
Richer					1.19*(1.04,1.36)
Richest ®					1.00
**Religion**					
Hindu					0.99(0.74,1.35)
Muslim					0.90(0.66,1.23)
Christian ®					0.92(0.64,1.33)
Others					1.00
**Caste**					
Scheduled Caste					0.99(0.88,1.13)
Scheduled Tribe					0.99(0.85,1.16)
Other Backward Class					0.96(0.86,1.06)
Others ®					1.00
**Region**					
North					1.47*(1.30,1.67)
Central					1.18*(1.05,1.31)
East					0.76*(0.67,0.86)
Northeast					0.74*(0.62,0.87)
West					1.21*(1.05,1.41)
South®					1.00
**Urban-ruralresidence**					
Urban					1.08(0.98,1.19)
Rural ®					1.00
Constant	0.19*(0.18,0.21)	0.13*(0.10,0.15)	0.11*(0.09,0.14)	0.10*(0.08,0.13)	0.08*(0.05,0.12)

Note: Model 1 includes IPI.

Model 2 includes IPI and maternal level variables.

Model 3 includes IPI, maternal level variables and child level variables.

Model 4 includes IPI, maternal level variables, child level variables and maternal and child care program variables.

Model 5 includes IPI, maternal level variables, child level variables and maternal and child care program variables and household level variables.

A number of control variables were also associated with LBW. The risk of LBW was higher among births of shorter mothers compared with taller mothers (Odds ratio – 1.34; 95% CI: 1.21 to 1.48). The risk of LBW was also significantly higher among births whose mothers had schooling up to secondary compared with births among mothers who had higher schooling. Preterm births were 2.16 (95% CI:1.92 to 2.42) times as likely as other births to be LBW in our study. Births to mothers who consumed less than 100 IFA tablets/equivalent syrup and received less than 2 TT injections were 1.18 (95% CI: 1.09 to 1.29) times and 1.20 (95% CI: 1.11 to 1.31) times more likely to be LBW compared with their counterparts respectively. Compared to the south, risk of LBW babies was higher in the north, central, and west regions of India, and lower in the east and northeast regions.

To examine the differential effect of IPI across birth order, mother's schooling, TT injections, and consumption of IFA tablets/syrup, stratified analyses were carried out (Table 3). In the stratified analyses, IPI less than six months was associated with LBW births only among higher birth orders (i.e. 3 or 4). Similarly IPI less than six months showed significant effects only among births to mothers who had no schooling and who had schooling up to primary. Likewise, the associations between IPI and LBW births were significant among those mothers who did not receive the recommended two or more TT injections or consumed 100 or more IFA tablets/equivalent syrup during pregnancy. Note that the associations between IPI and LBW were stronger in higher order births, non-literate or primary educated mothers, and mothers who did not receive the recommended TT injections and IFA tablets/equivalent syrup compared with the association seen in the overall analysis.

**Table 3:** Results of multivariable regression analysis for examining the association between IPI and LBW stratified across birth order, mother's schooling, TT injections and consumption of IFA tablets/syrup.

	Interpregnancy interval								
	<6	06–11	12–17	18-23 ®	24–29	30–35	36–41	42–47	≥48
	OR (95% CI)	OR(95% CI)	OR (95% CI)		OR(95% CI)	OR (95% CI)	OR (95% CI)	OR (95% CI)	OR(95% CI)
**Birth Order**									
1	1.08(0.67,1.75)	1.24(0.76,2.03)	1.35(0.81,2.26)	1	1.35(0.66,2.78)	1.60(0.70,3.67)	3.58*(1.03,12.44)	1.03(0.20,5.31)	0.07*(0.01,0.74)
2	1.20(0.99,1.45)	1.04(0.88,1.21)	1.11(0.96,1.27)	1	0.98(0.83,1.17)	1.09(0.82,1.45)	1.04(0.82,1.32)	1.09(0.75,1.57)	0.72(0.42,1.21)
3	1.32*(1.03,1.70)	1.11(0.89,1.39)	1.01(0.83,1.22)	1	1.10(0.87,1.39)	0.94(0.71,1.25)	0.86(0.60,1.24)	0.99(0.63,1.58)	2.37*(1.10,5.12)
≥4	1.49*(1.15,1.93)	1.01(0.80,1.28)	1.05(0.86,1.28)	1	1.14(0.89,1.45)	0.93(0.70,1.24)	0.99(0.69,1.45)	1.42(0.87,2.30)	1.89(0.85,4.17)
**Mother's Schooling**									
No schooling	1.35*(1.11,1.64)	1.14(0.96,1.36)	1.07(0.91,1.25)	1	1.10(0.91,1.33)	0.94(0.75,1.17)	0.96(0.72,1.28)	0.97(0.61,1.54)	2.23*(1.15,4.32)
Primary	1.42*(1.05,1.93)	1.17(0.90,1.51)	1.19(0.94,1.51)	1	1.26(0.94,1.67)	1.59(0.98,2.58)	1.30(0.84,1.99)	1.85(0.97,3.52)	1.43(0.48,4.28)
Secondary	1.06(0.87,1.28)	1.04(0.89,1.22)	1.08(0.93,1.25)	1	0.99(0.83,1.19)	0.95(0.75,1.20)	0.94(0.72,1.22)	1.01(0.69,1.45)	0.62(0.35,1.08)
Higher	1.10(0.67,1.82)	0.79(0.49,1.27)	0.93(0.60,1.44)	1	0.74(0.44,1.25)	0.84(0.43,1.62)	1.24(0.58,2.64)	1.08(0.41,2.87)	0.64(0.16,2.58)
≥2 TT **injections**									
No	1.42*(1.07,1.88)	1.02(0.79,1.31)	1.02(0.81,1.29)	1	1.09(0.82,1.44)	0.81(0.57,1.16)	1.15(0.77,1.71)	1.04(0.54,1.97)	0.91(0.34,2.40)
Yes	1.14(0.99,1.32)	1.08(0.95,1.22)	1.10(0.98,1.23)	1	1.04(0.91,1.19)	1.09(0.88,1.36)	1.01(0.82,1.22)	1.15(0.87,1.51)	1.32(0.85,2.06)
**Consumed** ≥100 IFA **tablets/equivalent syrup**									
No	1.29*(1.13,1.48)	1.10(0.98,1.25)	1.12*(1.01,1.25)	1	1.09(0.96,1.24)	0.97(0.82,1.14)	1.10(0.89,1.35)	1.03(0.75,1.40)	1.67*(1.02,2.73)
Yes	0.99(0.74,1.30)	0.97(0.76,1.24)	1.01(0.81,1.23)	1	0.95(0.73,1.23)	1.20(0.78,1.84)	0.84(0.60,1.19)	1.26(0.81,1.97)	0.62(0.33,1.16)

Note. Regression results are adjusted for other control variables listed in Table 1.

## 4 Discussion

Using data on 52,825 most recent births record as part of NFHS-4, our study findings indicate that short IPI (<6 months) is associated with a higher risk of LBW births in India. This finding holds even after adjusting for the well-known confounding factors such as components of ANC, preterm birth, parity, wantedness of birth, outcome of previous pregnancy, mother's tobacco consumption, etc. This finding is important given that 12% of recent births in India are accompanied with an IPI of less than 6 months. This means that about 3.2 million births in India are accompanied with an IPI of less than 6 months annually. The findings also indicate that the association between shorter IPI and LBW births is stronger among higher order births and mothers having no schooling or up to primary schooling. There is therefore a clear need to focus on women with shorter IPIs.

The association between short IPI and LBW in our study is in line with other research from diverse settings including the USA, Canada, Poland, Brazil, and Uruguay, which was conducted using live birth certificates, vital statistics and birth registries (Adams et al., 1997; Cecatti et al., 2008; Chen, Jhangri, Lacasse, Kumar, & Chandra, 2015; Cofer et al., 2016; Conde-Agudelo, Belizán, Norton, & Rosas-Bermúdez, 2005; Merklinger-Gruchala, Jasienska, & Kapiszewska, 2015; Zhu, Rolfs, Nangle, & Horan, 1999; Zhu, Haines, Le, McGrath-Miller, & Boulton, 2001; Zhu & Le, 2003). Our study found that an IPI of 48 months or more was not associated with higher risk of LBW births. Available evidence on the association between long IPI and the risk of LBW births is mixed. While some studies show that long IPI is significantly associated with a higher risk of LBW births (Conde-Agudelo et al., 2005; Qin et al., 2017; Zhu & Le, 2003), a few others report no association (Basso, Olsen, Knudsen, & Christensen, 1998; Klebanoff, 1988).

We also find significant association between the mothers’ recommended consumption of IFA tablets/equivalent syrup and LBW births. This finding is in line with the only study from India that, using pooled data from NFHS 1998-99 (NFHS-2) and NFHS 2005-06 (NFHS-3), also found significant association between consumption of IFA tablets/equivalent syrup and LBW births in India (Balarajan, Subramanian, & Fawzi, 2013). This is important given that the use of spacing methods of family planning, an important intervention to increase IPI, is currently limited in India. Only 11% of currently married women age 15–49 reported using a spacing method of family planning in NFHS-4. The use of highly effective spacing methods such as IUD/PPIUD (1.5%) and pills (4.1%) remains very low in the country. Moreover, use of spacing methods of family planning in India has not changed in the 10 years between NFHS-3 (2005-06) and NFHS-4 (2015-16) (IIPS & ICF, 2017). In addition, the stratified analysis by recommended IFA indicates that IPI less than 6 months was associated with LBW births only among mothers who did not consume the recommended IFA tablets/equivalent syrup. Likewise, the stratified analysis by recommended TT injections indicates that IPI less than 6 months was associated with LBW births only among mothers who did not receive the at least two TT injections. These findings indicate that increasing the coverage of recommended IFA and TT is likely to offset the adverse consequences of short IPI in India. Pregnant women must be encouraged to consume IFA tablets/equivalent syrup for at least 100 days. NFHS-4 indicates that seventy-eight percent of all women with a birth in the past five years were given or purchased iron and folic acid (IFA) tablets during the pregnancy for their most recent birth. But only 30% consumed the tablets for at least 100 days (IIPS & ICF, 2017).

Another finding that deserves mention is the relationship between short maternal height and higher risk of LBW births. The relationship between maternal height and the risk of LBW births shows an intergenerational effect of mother's nutritional status. Short stature of mother reflects on the poor nutritional status of mother during childhood and adolescence (Perkins, Subramanian, Davey Smith, & Özaltin, 2016; Steckel, 1995). A number of studies from the past have also shown a positive effect of maternal height on child health outcomes (Addo et al., 2013; Subramanian, Ackerson, Davey Smith, & John, 2009). This finding calls for greater attention to shorter pregnant women during the antenatal visits. Recently, The United Nations System Standing Committee on Nutrition suggested improving maternal nutrition for short-statured women, through improvements in preconception or conception diet quality, to break the intergenerational cycle of growth faltering in utero leading to poorer child health outcomes (UNSCN, 2010).

Our study has a number of strengths. A key strength of our study is the estimation and use of IPI in explaining LBW births in India. A majority of the previous studies from India have examined the effect of birth interval on LBW births (Deshmukh, Motghare, Zodpey, & Wadhva, 1998; Dhar, Shah, Bhat, & Butt, 1991; Kumar & Singhi, 1992). Such a definition ignores those pregnancies that resulted in adverse pregnancy outcomes such as miscarriage, abortion, and stillbirths between these two consecutive live births. Previous studies have reported a strong association between previous pregnancy outcomes and pregnancy outcomes in the next pregnancy (DaVanzo et al., 2004). For these reasons, the previous studies, using birth interval instead of IPI, might have overestimated the association between IPI and LBW births. Another key strength of our study is the availability of a nationally representative large-scale reproductive calendar on pregnancy history of women and birth weight for recently born children. The only study from India used hospital-based data for examining the association between IPI and risk of LBW births in Ahemadabad (Mavalankar et al., 1992). Hospital-based data usually suffer from serious selection bias. In hospital-based data, bias may also arise due to the overrepresentation of women who experienced any pregnancy-related complications. Such a bias may seriously modify the association between IPI and LBW births (Wendt, Gibbs, Peters, & Hogue, 2012). Fortunately, our data is not prone to such biases.

Limitations of our study may be noted. First, a selection bias may arise in our analysis due to the unavailability of reports of birthweight for about 22% of the recent births (IIPS & ICF, 2017). Children whose birthweight was not reported are more likely to belong to less educated mothers, poorest wealth quintile, home-based births, and living in rural areas compared with children of educated mothers, belonging to richer or richest wealth quintiles, born in a health facility, and living in urban areas (IIPS & ICF, 2017). Exclusion of these births, who are more likely to be LBW, may underestimate the association between IPI and LBW births. Therefore, our estimate of the association between IPI and LBW births can be safely taken as a lower bound to the true association.

Additionally, only about 55% of the available birthweights were recorded from the health card; the remaining 45% were based on mothers' recall. Therefore, there is a possibility of recall bias in 45% of the cases, though many studies rely on recall birthweight in a number of developing as well as developed countries (Lyall et al., 2016). To test this potential bias, we conducted a sensitivity analysis in which we analysed results separately for health card birthweights and for birthweights provided through mother's recall. Results from both groups were similar to that seen in the overall sample, albeit with slightly higher p-values for the recall group (health card OR = 1.27 for IPI<6 months vs. 18–23 months, p < 0.05; mother's recall OR = 1.16 for IPI <6 months vs. 18–23 months, p < 0.10). Moreover, in India, comparison of data across health cards and maternal recall reveals similar social patterning of low birthweight (Subramanyam, Ackerson, & Subramanian, 2010).

As our analysis is based on only those pregnancies that resulted in live births there is a possibility of selection bias, particularly given that short IPI is significantly associated with adverse pregnancy outcomes. Hence, excluding those pregnancies that resulted in non-live births may selectively reduce the population at risk of LBW in our sample, though as with missing birthweights, this bias will tend to make our estimates more conservative. We cannot rule out the possibility of recall bias in the reproductive calendar. Another limitation with the DHS reproductive calendar is that it provides the occurrence of an event in a particular month. However, event may occur at any date in the month. For example, if a pregnancy is terminated in the month of January and a woman becomes pregnant in the month of July, the IPI is 6 months. However, it is possible that the pregnancy was terminated on 31st January and woman became pregnant on 1st July, in which case the IPI would actually be 5 months. While we cannot rule out the possible effects of the absence of specific date reporting in the NFHS-4 reproductive calendar, we believe that the accuracy of this information would be overly compromised by recall bias over a five-year period. Due to the cross-sectional nature of the data we are unable to establish a causal relationship between IPI and LBW births.

Our findings provide compelling evidence on the association between short IPI and LBW births in India. Given the considerable prevalence of LBW births in India, there is an urgent need to increase the conception period following a live birth. Note that the mean and median IPI in India has not changed in the 10 years between NFHS-3 (2005-06) and NFHS-4 (2015-16). Focussing on promotion of spacing methods of family planning is an important strategy that India may consider. India's family planning programme has been a female sterilization driven programme, and the use of spacing methods has been limited. India was the first country in the world to launch an official family planning programme in 1952 (Ledbetter, 1984; Srinivasan, 1998). Although male and female sterilizations were introduced in the official family planning programme in 1966 (Gwatkin, 1979), male sterilizations was the dominant method through the late 1970s. Aggressive male sterilization camps were organized in the country during the late 1970s which led to a major shift in the method acceptance to female sterilization in the early 1980s (Basu, 1985; Gwatkin, 1979). Female sterilization has been the dominant method of family planning in India since then. Moreover, there has been a lack of focus on the spacing methods (Singh, 2018; Singh, Ogollah, Ram, & Pallikadavath, 2012). Despite the recommendation that every pregnant woman must be advised on family planning during the antenatal visits, only 69% of women who met with a community health worker in the last three months of pregnancy were advised on family planning (IIPS & ICF, 2017). There is thus a clear need to reposition spacing methods in the official Indian family planning programme. In addition, focus on norms change around family planning in general, and spacing family planning in particular is likely to help. Policymakers and programme managers should also make all efforts to provide high-quality recommended ANC services to all pregnant women irrespective of their socio-economic status. Studies indeed show significant socio-economic inequalities in the provision and utilization of recommended ANC in India in general and in rural areas in particular (Pallikadavath, Foss, & Stones, 2004; Pathak, Singh, & Subramanian, 2010; Singh, Pallikadavath, Ram, & Ogollah, 2012). Counseling women on the potential benefits of prolonged and exclusive breastfeeding during antenatal and postnatal care visits may help in delaying subsequent conception. In patriarchal societies like India, women have limited control over some aspects of their lives (Basu, 1996; Singh, Bloom, & Tsui, 1998; Stephenson & Tsui, 2002) and it is the husbands or mothers-in-law who play a dominant role in women's contraceptive decision-making (Singh et al., 1998). Therefore, the frontline health care providers should also counsel the husbands and other family members on the benefits of delaying the next pregnancy at the time of delivery in order to reduce the adverse pregnancy outcomes such as LBW births.

## Ethical statement

Our analysis is based on a publicly available dataset with no identifiable information in place. These data can be downloaded freely for research purposes from the MEASURE DHS website https://www.dhsprogram.com/what-we-do/survey/survey-display-355.cfm.

## Author statement

Ajit Kumar Kannaujiya (AKK) and Kaushalendra Kumar (KK) conceptualized the analysis; AKK and Ashish Kumar Upadhyay (AKU) conducted data analysis; Abhishek Singh (AS), KK and Lotus McDougal (LM) supervised the data analysis; AKK, AKU and KK wrote the first draft; AS and Anita Raj (AR) reviewed the first draft and edited the paper.

## Declaration of competing interest

None declared.
